# Prostatakarzinom-Screening? Nur evidenzbasiert, risikoadaptiert und organisiert!

**DOI:** 10.1007/s00103-024-03916-3

**Published:** 2024-08-05

**Authors:** Peter Albers, Nikolaus Becker

**Affiliations:** 1 Abteilung für Personalisierte Früherkennung des Prostatakarzinoms, Deutsches Krebsforschungszentrum (DKFZ) Heidelberg, C130, Im Neuenheimer Feld 581, 69120 Heidelberg, Deutschland; 2 Klinik für Urologie, Universitätsklinikum Düsseldorf, Medizinische Fakultät, Heinrich-Heine-Universität Düsseldorf, Düsseldorf, Deutschland

**Keywords:** Prostatakarzinom, Screening, PSA, Risikoadaptation, Implementierung, Prostate cancer, Screening, PSA, Risk-adapted, Implementation

## Abstract

Aufgrund einer Kommissionsempfehlung der Europäischen Union aus dem Jahr 2022, neue Screeningstrategien für das Bronchialkarzinom, das Magenkarzinom und das Prostatakarzinom zu entwickeln, ist auch Deutschland dazu aufgerufen, sein Prostatakarzinom-Früherkennungsprogramm zu überarbeiten. In diesem Diskussionsartikel wird eine Übersicht über neue Ergebnisse zur Prostatakrebsfrüherkennung gegeben, die auf die Empfehlung eines organisierten, risikoangepassten Prostatakrebsscreenings hinauslaufen. Basierend auf den ersten Resultaten der aktuellen deutschen Prostatascreening-Studie PROBASE und neuer internationaler Literatur sollten Modellprojekte begonnen werden, die die spezifischen Bedingungen für ein organisiertes risikoadaptiertes Prostatakarzinom-Screeningprogramm erkunden.

## Abstract (en)

In view of a recent recommendation of the European Commission to conceptualize novel screening approaches for lung, gastric, and prostate cancer, Germany is also invoked to revise its prostate early detection program. This discussion article provides an overview of new findings on prostate cancer screening, which suggest an organized and risk-adapted screening approach. Based on the German risk-adapted screening trial PROBASE, together with recently published data on organized screening programs in Europe, model projects should be established to determine the specific modalities for a new organized and risk-adapted prostate cancer screening program.

## Einleitung

Deutschland hat mit seinem „Gesetzlichen Früherkennungsprogramm“ aus dem Jahr 1971 als eines der ersten Länder mit Krebsfrüherkennung begonnen [1]. Bei der Einführung moderner Screeningverfahren wurde hierzulande bisher allerdings eher zögerlich verfahren. Nur mit dem organisierten Mammografiescreeningprogramm aus dem Jahr 2005 [2] gelang es, alle Anspruchsberechtigten durch postalisches Anschreiben auch tatsächlich zu erreichen, zu informieren und zur Teilnahme einzuladen. Für Gebärmutterhalskrebs und Darmkrebs wurde vor wenigen Jahren ein abgeschwächt organisiertes Screening eingeführt, bei dem die Anspruchsberechtigten lediglich schriftlich kontaktiert und informiert werden. Für das Prostatakarzinom gilt immer noch die Empfehlung aus dem Jahr 1971, sich ab dem Alter von 45 Jahren jährlich einer Tastuntersuchung zu unterziehen, obwohl für dieses Verfahren nie ein Wirksamkeitsnachweis erbracht wurde und umgekehrt wiederholt Belege für eine mangelnde Wirksamkeit vorgelegt wurden (jüngst wieder in [3]).

Starke Impulse zur Einführung oder Überarbeitung organisierter Screeningverfahren bezüglich der häufigeren Krebskrankheiten kommen von der Europäischen Union (EU; [4–6]). Im März 2022 hat sie nun die Mitgliedsstaaten dazu aufgerufen, Screeningempfehlungen auch für das Lungenkarzinom, das Magenkarzinom und ebenso das Prostatakarzinom auszusprechen oder zu überarbeiten [7]. Damit wären dann bis auf das Pankreaskarzinom für alle häufigen Krebsarten Früherkennungsmaßnahmen definiert.

In diesem Diskussionsartikel werden die Erkenntnisse aus den jüngst publizierten Screeningstudien zum Prostatakarzinom dargestellt und daraus Empfehlungen für ein zukünftiges Screeningprogramm in Deutschland abgeleitet.

## Evidenzbasiertes Prostatakarzinom-Screening

Die europäische randomisierte PSA-basierte Prostatakarzinom-Screeningstudie (European Randomized Study of Screening for Prostate Cancer – ERSPC) konnte einen 20 %igen krebsspezifischen Mortalitätsvorteil für das Screening bei einer hohen Rate von Überdiagnosen zeigen (162.243 Teilnehmer, 214 am Prostatakarzinom verstorbene Männer in der Screeninggruppe, 326 am Prostatakarzinom verstorbene Männer in der Kontrollgruppe; [8]). Das Prostatakarzinom ist der einzige Tumor, der durch einen einfachen Blutwert erkannt werden kann („prostata-spezifisches Antigen“– PSA). Das Institut für Wirtschaftlichkeit und Qualität im Gesundheitswesen (IQWiG) hat daraufhin zwar zu Recht festgestellt, dass der Schaden einer PSA-basierten Früherkennung selbst in einem organisierten Verfahren größer sei als der Nutzen [9]. Aber seit Mitte der 1990er-Jahre hat sich der selbstinitiierte PSA-Test als „opportunistisches Screening“ nicht nur in Deutschland verbreitet und für diese Art des Screenings trifft die ungünstige Schaden-Nutzen-Relation in noch höherem Maße zu [10]. Dieser scheinbare Widerspruch wird verständlich, wenn man sich die Problematik des potenziellen Schadens des Prostatakrebs-Screenings vor Augen führt. Um ihn einzudämmen, wurden in der Folge der ERSPC-Studie erhebliche wissenschaftliche Anstrengungen unternommen, die seit der Stellungnahme des IQWiG zu aussagekräftigen Publikationen geführt haben, die darauf hinauslaufen, dass durch ein durchdachtes Einsetzen des PSA-Werts in einem organisierten Screeningprogramm der Nutzen erhöht und der Schaden vermindert werden kann.

### Was sind Nutzen und Schaden eines Krebsfrüherkennungsprogramms?

Die auf den ersten Blick naheliegende Größe „Verlängerung der Überlebenszeit durch Screening“ als Maß für den Nutzen eines Screeningprogramms ist leider untauglich, weil durch die mit der Früherkennung zwangsläufig verbundene Vorverlagerung des Diagnosezeitpunktes das Überleben immer verlängert erscheint, selbst wenn der betreffende Patient am exakt selben Tag sterben würde wie ohne Früherkennung. Der Nutzen eines Screeningprogramms wird daher üblicherweise durch die mit dem Programm erzielte Senkung der krankheitsspezifischen Mortalität quantifiziert [1, 11]. Der Einfluss des Prostatakarzinom-Screenings auf die Gesamtmortalität ist schwer zu beweisen, da der Anteil dieser Krebsart an der Gesamtmortalität gering ist und bislang jedes Prostatakrebs-Screeningprogramm auch aufgrund der nur langsam progredienten, oft in hohem Alter entstehenden Erkrankung keinen oder einen nur sehr geringen Einfluss auf die Gesamtmortalität hat [12].

Da die relative Mortalitätsreduktion der bei Weitem effektivsten Krebsfrüherkennung, derjenigen des Zervixkarzinoms, von bis 62 % [13] für andere Krebsarten nicht im Entferntesten erreicht werden kann, wird der Nutzen eines organisierten Screenings von Kritikern mitunter als zu gering angezweifelt [14]. So wird die Programmeffektivität (Mortalitätsreduktion unter den zum Screening *eingeladenen* Personen) für das Mammografiescreening mit 25–30 % angegeben, ebenso für das Darmkrebsscreening.

Aus der erwähnten ERSPC-Studie wissen wir nach dem jüngst publizierten 21-Jahre-Follow-up, dass für die PSA-basierte Prostatakrebsfrüherkennung relative 27 % erreicht werden [15]. Anzumerken ist, dass in die Größe „Programmeffektivität“ die Teilnahmebereitschaft der anspruchsberechtigten Bevölkerungsgruppe eingeht und der Nutzen für die tatsächlichen Teilnehmer deutlich höher liegt, so beim Mammografiescreening bei 40–50 % relativer Mortalitätsreduktion, beim Darmkrebs bei 70 % und beim Prostatakrebs in der ERSPC-Studie bei 51 %, d. h. ähnlich zum Mammografiescreening. Allerdings bleibt zu erwähnen, dass die relativen Mortalitätsraten durch die Selektion der Teilnehmer falsch hoch sein können, wenn nur Personen an Screeningstudien teilnehmen, die ein hohes Gesundheitsbewusstsein haben („healthy screenee bias“).

Korrespondierend zur Reduktion der Mortalität konnte in der ERSPC-Studie eine um 32 % geringere Metastasierung bei den detektierten Tumoren beobachtet werden [15]. Die ERSPC-Studie hat Anfang der 1990er-Jahre die Rekrutierung begonnen. Damals konnten vornehmlich Knochenmetastasen diagnostiziert werden, die aber nur schlecht behandelbar waren. Daher war die Überlebenszeit mit Knochenmetastasen gering. Heutzutage können Metastasen eines Prostatakarzinoms gut behandelt werden und die so behandelten Patienten leben häufig mehr als 10 Jahre [16].

Allerdings führt die Behandlung des metastasierten Prostatakarzinoms zu erheblichen Einschränkungen der Lebensqualität in Verbindung mit sehr hohen Therapiekosten, sodass das Ziel der Metastasenfreiheit bei detektierten Tumoren sowie deren Behandlung in den Fokus der Prostatakrebsfrüherkennung rücken sollte. Zum Vergleich: Die Behandlung einer metastasierten Prostatakrebserkrankung kostet mehrere Hunderttausend Euro, die Primärbehandlung (Operation oder Strahlentherapie) lediglich ein Bruchteil (10.000–20.000 €; [17]).

Für die Entscheidung über die Sinnhaftigkeit des Aufbaus eines organisierten Screeningprogramms ist hinsichtlich des „Nutzens“ die Programmeffektivität *maßgeblich*. Da die systematische Suche nach Krebserkrankungen im Frühstadium auch unvermeidlich unerwünschte Nebeneffekte mit sich bringt, sozusagen einen „Schaden“ anrichtet, spielt die Balance zwischen „Nutzen“ und „Schaden“ für die Qualität und Rechtfertigung eines Screeningprogramms aber die *entscheidende* Rolle. Zu den unerwünschten Effekten gehört, dass der Screeningtest (Abstrich, Mammografie, Darmspiegelung, PSA-Test) nicht perfekt ist und auch bei Personen auffällig erscheint, bei denen sich keine Krankheit nachweisen lässt („falsch-positive“ Befunde). Angesichts des Screenings unter weitgehend „gesunden“ Anspruchsberechtigten (nur bei weniger als 1 % der betreffenden Personen wird sich je Teilnahmerunde eine Krebserkrankung zeigen) führen diese proportional gesehen seltenen Fälle zu einer mehr oder weniger hohen Zahl an „überflüssigen“ Abklärungsuntersuchungen. So liegt der Anteil der tatsächlich nachgewiesenen Mammakarzinome bei etwa 20 % der verdächtigen Mammografien, beim Prostatakrebs lag der Wert in der ERSPC-Studie bei ca. 23 % [15]. In der PROBASE-Studie hatten in der ersten Screeningrunde bei 45-Jährigen von 186 PSA-Screeningtest-auffälligen Probanden 120 eine Biopsie und letztlich 48 der Biopsierten (40 %) ein Prostatakarzinom [18]. Noch wichtiger erscheint aber in diesem Zusammenhang die mögliche Identifikation einer Gruppe niedrigen Risikos, bei der keine höherfrequenten Screeninguntersuchungen stattfinden müssen [19]. Beide Parameter variieren je nach Qualität des Screeningtests und der Folgeuntersuchungen und stellen damit eine der Schlüssel-„Stellschrauben“ für die Effektivität und die Kosten des Screeningprogramms dar.

Insgesamt sind folgende Parameter für die Effektivität eines zukünftigen Screeningprogramms entscheidend: das Alter zu Beginn des Screenings (und das Alter am Ende des Screeningprogramms), eine moderne Abklärungsstrategie (basierend auf einem Basis-PSA-Wert und weiteren serum-/urinbasierten Biomarkern und der Magnetresonanztomografie) sowie entsprechend reduzierte Screeningintervalle für Männer ohne oder mit nur sehr geringem Risiko für ein Prostatakarzinom [20–23].

Der größtmögliche Schaden der Früherkennung ist schließlich die Detektion von Karzinomen, die zu Lebenszeit der betreffenden Personen nie in Erscheinung getreten wären („Überdiagnose“) und erst recht nicht hätten behandelt werden müssen („Übertherapie“). Überdiagnose ist bis zu einem gewissen Grad unvermeidlich und erreicht bei den verschiedenen Krebsarten ein unterschiedliches Ausmaß. Die Zahlen reichen von 10 % beim Mammakarzinom bis zu deutlich höheren Werten beim Prostatakarzinom, für die je nach Alter und PSA-Wert ein Bereich von 2 % bis über 50 % angegeben wird [12, 24–27].

In diesem Zusammenhang können Prostatakarzinome in niedrigem Stadium mit niedriger Aggressivität (pT1c, ISUP-GG 1) als Karzinome angenommen werden, die entsprechend den Daten der ProtecT-Studie lange Zeit ohne Progression bleiben [28]. Je älter die Screeningkohorte ist, desto höher ist dieser Anteil unnötig detektierter Tumoren. Ob diese beschriebene Annahme auch für die jüngere Altersgruppe der z. B. in PROBASE diagnostizierten Karzinome zutrifft, muss noch gezeigt werden. Insgesamt begründet die hohe Rate der durch Screening detektierten Karzinome geringer Aggressivität die erwähnte Zurückhaltung vieler Länder bei der Einführung eines Prostatakrebsscreenings und die negative Beurteilung seitens des IQWiG. Bei dieser ungünstigen Ausgangssituation kann in der Erhöhung des Anteils an verzögerter Therapie beim Prostatakrebs mit niedriger Aggressivität eine weitere essentielle „Stellschraube“ gesehen werden. Durch die konsequente Anwendung der aktiven Überwachung würden Effektivität und Kosten eines Screeningprogramms positiv beeinflusst werden. Und hier setzten auch die oben erwähnten Forschungsanstrengungen an, deren Ergebnisse zu einer markant anderen Beurteilung der Wertigkeit eines Prostatakarzinom-Screenings geführt haben.

In diesem Zusammenhang ist eine objektive Information über Vor- und Nachteile sowohl des Screenings als auch der daraus resultierenden unterschiedlichen Therapieoptionen vor allem aus Sicht der behandelten Patienten („patient-reported outcome“) vor Beginn des Screenings notwendig [29, 30].

Die durch „falsch-positive“ Befunde und durch begrenze Mortalitätsreduktion gegebenen Eckdaten eines Screeningprogramms lassen sich mit den folgenden Fragen erheben:

- Wie viele Personen müssen gescreent werden?
- Wie viele Befunde sind auffällig und müssen abgeklärt werden?
- Wie viele Tumoren werden verifiziert und wie viele Auffälligkeiten sind „falsch-positiv“?
- Wie viele krankheitsbedingte Todesfälle treten trotz Screenings auf?
- Wie viele krankheitsbedingte Todesfälle werden vermieden?
- Wie viele Krebsdiagnosen wurden dabei „überdiagnostiziert“?

Bei etwa gleich hoher Rate an auffälligen Befunden unterscheidet sich das Mammografie- vom Prostatascreening vor allem in der hohen Rate aggressiver Tumore [31, 32].

## Das risikoadaptierte Prostatakarzinom-Screening

### Der PSA-Basiswert

Neuere Ergebnisse einer schwedischen Studie deuten darauf hin, dass ein bei Männern mittleren Alters bestimmter PSA-Wert einen hohen prädiktiven Wert hinsichtlich des lebenslangen Erkrankungsrisikos für Prostatakrebs hat [33]. Dieses Ergebnis lässt sich dazu nutzen, die Gesamtzahl durchzuführender Screeningtests und damit die Zahl und die Kosten falsch-positiver Befunde und unnötiger Abklärungsuntersuchungen zu senken. Ausschlaggebend hierfür ist, dass der PSA-Wert dann bestimmt wird, wenn die Prostatadrüse noch nicht gutartig vergrößert ist, also etwa im Alter bis etwa 50 Jahre. Die PROBASE-Studie, die dieses Konzept prospektiv testet, konnte zeigen, dass nur etwa 1–2,5 % der Teilnehmer über dem als Schwellenwert für Abklärungsbedarf definierten Wert von 3,0 ng/ml liegen [18]. Zum Vergleich: Im Alter von 55–69 Jahren ist der Anteil der über diesem Schwellenwert liegenden PSA-Werte 20-mal höher und damit auch Aufwand und Kosten von Folgeuntersuchungen. Die Kernbotschaft der schwedischen Studie ist aber, dass der in mittlerem Alter bestimmte Wert eine prädiktive Kraft hat, d. h., bei einem in diesen vergleichsweise jungen Jahren erhöhten PSA-Wert finden sich nicht nur mehr Karzinome, sondern es ist auch die Wahrscheinlichkeit erhöht, innerhalb der nächsten 25 Jahre ein metastasiertes Prostatakarzinom zu bekommen [33]. Daraus lässt sich eine risikoangepasste Screeningstrategie entwickeln.

### Die risikoadaptierte Diagnostik

Die erwähnte schwedische Studie fand heraus, dass Männer, deren Basiswert unter dem Median der in der Altersgruppe der 45- bis 50-jährigen Männer gemessenen PSA-Werte lag (bei 45- bis 49-Jährigen 0,68 ng/ml), ein extrem geringes Lebenszeitrisiko hatten (0,09 %), ein metastasiertes Prostatakarzinom zu entwickeln [33]. In einer prospektiven englischen Studie, der Vorläuferstudie von ProtecT, konnte gezeigt werden, dass die Prävalenz für ein Prostatakarzinom bei jungen Männern (45–49 Jahre) bei 2,3 % lag, wenn der „Cut-off“ für eine Biopsie bei einem PSA-Wert von 1,5 ng/ml lag [34]. Auf diesen Daten basiert die Risikoklassifikation in der PROBASE-Studie (Tab. 1). Als Konsequenz dieser Risikogruppeneinteilung werden in einem risikoadaptierten Screening Männer im Alter von 45 Jahren in der Niedrigrisikogruppe (PSA < 1,5 ng/ml) nur noch alle 5 Jahre erneut PSA-getestet. Hierzu konnte die PROBASE-Studie unterstützende Daten zeigen. Nur 0,4 % der Männer in dieser Gruppe hatten 5 Jahre später einen erhöhten PSA-Wert > 3,0 ng/ml und es entwickelten sich in dieser Gruppe insignifikant mehr Karzinome [19]. Sobald der PSA-Wert > 3,0 ng/ml liegt, erfolgt eine spezielle Prostatabildgebung durch eine multi- oder biparametrische Magnetresonanztomografie (MRT). Durch diesen Reflextest kann die Rate der Männer, die sich einer invasiven Diagnostik unterziehen müssen, um 30–40 % reduziert werden [35–37]. Ob zusätzliche blutbasierte Biomarker die Rate an MRTs weiter reduzieren können, wird sich in zukünftigen Studien, wie z. B. ProScreen aus Finnland, zeigen [38].

**Tab. 1:** Risikokategorisierung aufgrund des Basis-PSA-Werts mit 50 Jahren [18]

Basis-PSA-Wert	Risikogruppe	Folgeuntersuchung nach
< 1,5 ng/ml	Niedriges Risiko	5 Jahren
1,5–2,99 ng/ml	Mittleres Risiko	2 Jahren
≥ 3,0 ng/ml	Hohes Risiko	MRT und bei PIRADS 3–5 Biopsie

*MRT* multiparametrische Magnetresonanztomografie, *PIRADS* Prostate Imaging Reporting and Data System

Selbst die Detektion des Tumors wird durch die MRT erleichtert, denn in der Göteborg-Studie hat es ausgereicht, nur die auffälligen MRT-Läsionen zu biopsieren und nicht mehr die gesamte Prostata. Dies hat in dieser Studie zur deutlichen Reduktion der Rate an Überdiagnosen geführt, obwohl sicher ein geringer Teil der klinisch signifikanten Karzinome durch diese Strategie verzögert diagnostiziert wurden. Ob diese guten Daten auch in der zukünftig zu präferierenden Screeningaltersgruppe der 45- bis 50-Jähren zu bestätigen sind, wird gerade in der PROBASE-Studie untersucht. Erste Ergebnisse deuten darauf hin, dass bei einem durch Experten befundeten MRT ähnlich gute Ergebnisse erzielt werden können [39].

Ob die risikoadaptierten Screeningmodelle tatsächlich zu einer Reduktion der Überdiagnose führen, hängt von unterschiedlichen Parametern, wie z. B. der Bewertung von ISUP-GG 2‑Karzinomen, und der Detektionsrate in weiteren Screeningrunden ab und kann in Modellierungen kalkuliert werden [40, 41].

### Aktive Überwachung von Prostatakarzinomen

PROBASE konnte zeigen, dass die überwiegende Zahl der im Screening gefundenen Karzinome wenig aggressiv war (ISUP-GG 1 und 2). Bei älteren Patienten wurde in der ProtecT-Studie bewiesen, dass das aktive Abwarten mit regelmäßigen PSA-Kontrollen der sofortigen Operation oder Bestrahlung mit einer krebsspezifischen Mortalität von jeweils unter 3 % nach 15 Jahren gleichwertig war [28]. Diese Studie hat die Sicht auf die Therapie des Prostatakarzinoms grundlegend verändert und könnte bei konsequenter Anwendung die Nebenwirkungen einer unnötigen oder zu frühen Therapie bei gleicher Effektivität drastisch reduzieren.

### Kosteneffizienz des modernen PSA-basierten Screenings

Eine der aktuell wichtigsten Nachrichten der PROBASE-Studie ist, dass bei früher Bestimmung des Basis-PSA-Werts im Alter von 45–50 Jahren ca. 90 % der Männer der Gruppe mit niedrigem Risiko für PSA-Anstiege und die Entwicklung eines Prostatakarzinoms zugeordnet werden können (PSA < 1,5 ng/ml; [18]). Sollte sich diese Entwicklung weiter bestätigen, dann entspräche sie den schwedischen Ergebnissen, dass keine wie bislang jährliche Wiederholung des Screenings erforderlich ist. Anders ausgedrückt, das moderne PSA-Screening würde nur für 10 % der Männer mit 45–50 Jahren bedeuten, dass sie häufiger untersucht bzw. diagnostiziert werden müssten. In der Kombination dieses risikoadaptierten Screenings mit hoher Sensitivität zur frühen Entdeckung aggressiver Prostatakarzinome, der Therapieverzögerung oder Therapievermeidung durch aktive Überwachung und durch die Reduktion der Screeninggruppe auf nur 10 % durch eine Einmalbestimmung des PSA-Werts im richtigen Alter könnte das Prostatakrebs-Screening sehr effektiv und damit auch kosteneffizient werden.

## Das organisierte Einladungsverfahren – Notwendigkeit von Implementierungsstudien

Die Akzeptanz selbst hervorragend sensitiver Screeningmethoden, wie z. B. der Koloskopie zur frühen Entdeckung und auch frühen Therapie des kolorektalen Karzinoms, ist in Deutschland leider nicht hoch, sie liegt bei etwa 30 %. Das Mammografiescreening wird von etwa 50 % der Frauen akzeptiert. Als Surrogatparameter für die Akzeptanz eines risikoadaptierten Screeningprogramms konnte man in PROBASE feststellen, dass fast 80 % der Studienteilnehmer in der Niedrigrisikogruppe wirklich 5 Jahre keine selbstinitiierten PSA-Tests durchführen [42]. In Schweden wird aktuell seit 2020 eine nationale Implementierungsstudie durchgeführt, die in ersten Auswertungen eine Akzeptanz und Beteiligungsrate von ca. 35 % aufweist [43, 44]. Vieles kann hier eine gute Aufklärung über die aktuellen Zahlen und Ziele eines nationalen Screeningprogramms bewirken. Andere in Europa aktuell geprüfte Implementierungsstudien werden über das Programm PRAISE‑U im Rahmen des EU4Health-Programms koordiniert und werden in den nächsten Jahren wichtige Hinweise für die Einführung von nationalen Prostatascreeningstrategien liefern [45].

In Deutschland ist eine Implementierungsstudie notwendig, bevor ein organisiertes Prostatascreeningprogramm gestartet wird. Zunächst muss der am besten akzeptierte Zugang zum Basis-PSA-Test untersucht werden (Blutabnahme über Hausarzt, Urologe, Labor oder als Selbsttest). Des Weiteren müssen die Eingeladenen in einem Screeningregister erfasst sein, um die Risikogruppen in unterschiedlichen Zeitintervallen wieder einladen zu können. Und letztlich müssen die Qualität und Verfügbarkeit der Diagnostik, z. B. durch MRTs, geprüft sein.

## Ausblick und Fazit

Mit einem geeigneten Einsatz des PSA-Tests kann die bisher eher negativ gesehene Nutzen-Schadens-Bilanz eines Prostatakrebs-Screenings mit dem PSA-Test deutlich in Richtung eines überlegenen Nutzens verschoben werden. Das heißt: Die krebsspezifische Mortalität kann gesenkt und das metastasenfreie Leben nach einer Prostatakrebsdiagnose verlängert werden. Zur Minimierung der negativen Effekte ist die Prostatakrebsfrüherkennung in Form eines modernen bevölkerungsbezogenen Screeningprogramms auszurollen, das folgende Eigenschaften aufweisen sollte:

1. Das Programm muss „organisiert“ sein, d. h., die Anspruchsberechtigten müssen zum gegebenen Zeitpunkt (z. B. im Alter von 50 Jahren) angeschrieben, informiert und auch gezielt zum Screening eingeladen werden; nur so ist garantiert, dass der lebenslang wichtige Basiswert zum richtigen Zeitpunkt erhoben wird und die aus der Risikoadaption sich ergebenden Screeningintervalle zur Verminderung überflüssiger Screeningtests und damit einhergehender falsch-positiver Befunde eingehalten werden.
2. Bei auffälligen Screening-PSA-Tests sind zur weiteren Verminderung falsch-positiver Befunde und überflüssiger Biopsien konfirmatorische PSA-Tests und/oder MRTs durchzuführen, um damit die Programmakzeptanz zu erhöhen.
3. Bei nicht auffälligen Screening-PSA-Tests muss den Teilnehmern des Screeningprogramms verlässlich vermittelt werden, dass weitere Tests erst nach einigen Jahren erforderlich sind.
4. Die Information der Anspruchsberechtigten sollte von Anfang an darauf hinweisen, dass – im Unterschied zu vielen anderen Krebsarten – nicht jede Prostatakarzinomdiagnose zwangsläufig zur sofortigen Entfernung des Tumors im Rahmen belastender, die Lebensqualität einschränkender invasiver Behandlungen führen muss. Nicht aggressive Karzinome sollten zunächst aktiv überwacht werden, bis sich klare Indikationen für die Einleitung therapeutischer Maßnahmen ergeben. Auch das Sichtbarmachen einer zurückhaltenden Behandlungsstrategie könnte die Akzeptanz eines Prostatakrebsscreenings erhöhen.

Die Effektivität eines Prostatakrebs-Screenings mit PSA-Tests wurde in randomisierten Studien zweifelsfrei nachgewiesen und muss nicht noch einmal gezeigt werden. Ob die Nutzen-Schadens-Bilanz eines darauf aufbauenden bevölkerungsbezogenen Screeningprogramms eher positiv oder eher negativ ausfällt, ist eine Sache der praktischen Durchführung, in der die oben als „Stellschrauben“ bezeichneten Parameter mehr oder weniger effektiv genutzt werden. Dabei sollte man auf die ersten Erfahrungen in anderen Ländern (z. B. OPT-Programm in Schweden) zurückgreifen und für Deutschland spezifische, organisierte Programme testen. Vorstellbar wären vor allem Bestrebungen, die PSA-Testung als Eingangsuntersuchung und Basis eines risikoadaptierten Screeningprogramms zu finanzieren (GKV) und erst im Falle einer notwendigen Diagnostik Fachärzte zu involvieren. Hierbei muss insbesondere die Qualität der Folgeuntersuchungen (MRT und MRT-gestützte Prostatabiopsie) im Vordergrund stehen. Signifikante Reduktionen des Schadens eines Screeningprogramms hängen von der Qualität der darin verwendeten Spezialuntersuchungen ab. Denkbar wären hier auch spezialisierte Screeningzentren oder externe Qualitätskontrollen, z. B. über telemedizinische MRT-Befundung oder die Anwendung maschinenerlernter MRT-Befunde (künstliche Intelligenz).

Zur Entwicklung eines solchen Programms könnten die aktuellen Empfehlungen der EAU, die auch in den aktuellen PRAISE-U-Studien (www.praise-u.eu) geprüft werden, die Grundlage eines organisierten Screenings darstellen. Prüfparameter wären aber die optimale Information der Bevölkerung (Kommunikationsstrategie), der optimale Zugang zum Screening und die Qualität der Diagnostik (MRT, MRT-gestützte Biopsie). Eine Implementierungsstudie zur Testung dieser für Deutschland relevanten Prüfparameter wäre unbedingt notwendig, bevor ein organisiertes Screeningprogramm aufgesetzt wird.
